# In Silico Prediction of Secreted Proteins in Shiga Toxin-Producing *Escherichia coli*: Identification of a Hydrolase as a Robust Genomic Marker

**DOI:** 10.3390/vetsci13020153

**Published:** 2026-02-04

**Authors:** María Victoria Vélez, Ana Elisa Juárez, Rocío Colello, Felipe Del Canto, Nora Lía Padola

**Affiliations:** 1 Laboratorio de Inmunoquímica y Biotecnología, Centro de Investigación Veterinaria de Tandil (CIVETAN), UNCPBA-CICPBA-CONICET, Facultad de Ciencias Veterinarias, Tandil 7000, Argentina; mvictoriavelez@vet.unicen.edu.ar (M.V.V.); anajuarez@vet.unicen.edu.ar (A.E.J.); rocioc@vet.unicen.edu.ar (R.C.); 2 Núcleo Interdisciplinario de Microbiología, Instituto de Ciencias Biomédicas, Facultad de Medicina, Universidad de Chile, Santiago 8320000, Chile

**Keywords:** proteins, STEC, novel diagnostic, possible therapeutic targets, hydrolase

## Abstract

Shiga toxin-producing *Escherichia coli* (STEC) are major foodborne pathogens that can cause severe human disease. Identifying molecular markers associated with pathogenicity is essential to improve detection and to better understand virulence mechanisms. In this study, 35,828 *E. coli* genomes available in a public database were analyzed with the purpose of identifying STEC genes encoding proteins secreted to the outer membrane or into the extracellular space. The strains belong to six different sequence types (STs): ST442, ST297, ST1131, ST2217, ST2387, and ST2520. Comparative genomics allowed identification of 155 genes that were initially evaluated as potential virulence-associated candidates. Among these, a hydrolase-encoding gene showed a wide distribution and a significant association with STEC genomes versus non-STEC genomes (81.6% in the STEC group and 15.8% in the non-STEC group). The protein product is predicted to be released into the extracellular space and, according to examples in other bacterial pathogens, may contribute to STEC virulence. Altogether, our findings highlight this hydrolase as a relevant molecular marker that could complement current genetic screening schemes and support the design of novel control strategies against STEC infections.

## Simple Summary

Shiga toxin-producing *Escherichia coli* is a foodborne bacterium that can cause severe illness in humans. Identifying harmful strains remains challenging due to their high genetic and biological diversity. In this study, we analyzed more than thirty-five thousand *Escherichia coli* genomes from public databases using computer-based methods to identify disease-associated factors. We focused on proteins secreted outside the bacterial cell as potential markers. One hundred fifty-five candidate genes were identified, and a hydrolase-encoding gene showed a strong association with pathogenic strains. This hydrolase could complement existing screening methods and support the development of improved strategies for detection and control.

## 1. Introduction

*Escherichia coli* (*E. coli*) is a widespread bacterium that inhabits the intestinal tract of humans and food-producing animals as part of their normal microbiota [1,2]. Most *E. coli* strains are harmless commensals that coexist with their hosts in a mutualistic relationship and rarely cause disease [3]. However, this species displays remarkable genetic and phenotypic diversity, giving rise to pathogenic variants capable of causing a wide range of infections. Taking into account the virulence factors they harbor and the clinical manifestations they cause, Shiga toxin-producing *E. coli* (STEC) has gained relevance. STEC constitutes a zoonotic food-borne pathogen that causes diarrhea and Hemolytic Uremic Syndrome (HUS) [4,5]. Cattle are recognized as the principal reservoir of STEC, excreting the pathogen in their feces [6]. This ecological relationship allows STEC to persist in the bovine gastrointestinal tract as commensal organisms, facilitating their release into the environment through intermittent or continuous shedding [7,8]. Consequently, human infection can occur through multiple exposure pathways, such as the consumption of undercooked meat, contaminated water -of particular concern given its role in the contamination of fresh produce- as well as through direct contact with colonized animals or their environments [9,10].

Shiga Toxin (Stx) is the defining virulence factor of STEC. Stx is categorized into two main groups, *stx1* and *stx2*, which are further divided into numerous subtypes [11,12]. A single STEC strain may produce one or multiple Stx subtypes. Notably, strains producing Stx1a, Stx2a, and Stx2d are primarily associated with severe diarrhea and HUS [13,14].

Although Shiga toxins (Stx) are recognized as the principal virulence factor responsible for the pathogenicity of STEC, additional determinants are likely to contribute to the development of disease [15,16]. To exert their toxic effects, bacteria must first adhere to the host gastrointestinal tract [17]. The Locus of Enterocyte Effacement (LEE) encodes genes involved in intestinal epithelial adherence and the formation of attaching and effacing lesions [18]. Nevertheless, LEE-negative STEC strains have also been isolated from clinical cases, suggesting the existence of alternative and poorly understood adherence mechanisms, and little is known about their virulence strategies toward epithelial cells [19].

Additional virulence factors, such as outer membrane proteins (OMPs), display a broad range of functions, acting as adhesins for bacterial infection [20], passive diffusion and efflux channels [21], siderophore receptors [22], protein translocation pores [23], and enzymes [24,25]. Secreted and surface-exposed proteins represent optimal targets for vaccine and diagnostic applications, as they constitute the primary interface between the pathogen and the host immune system [26,27]. The identification and characterization of additional virulence factors are essential to elucidate STEC pathogenic mechanisms and may contribute to identifying effective strategies to prevent transmission [28]. Various studies have reported successful approaches to reduce STEC shedding and spread, including vaccination of cattle using specific antigens derived from LEE-encoded virulence factors [27]. However, their limited coverage underscores the need for alternative antigens that can provide broader protection across STEC, which can protect against a broader range of serotypes. To this end, our objectives were to detect and characterize proteins in silico and to evaluate their potential as specific markers for STEC identification.

## 2. Materials and Methods

### 2.1. The Strains Selections

The bacterial strains were selected from the strain collection of the Laboratory of Immunochemistry and Biotechnology, FCV, UNCPBA, Argentina, for which whole-genome sequence data are available [28]. Genomes of 11 LEE-negative STEC strains were analyzed. The bacteria were isolated from cattle, and they harbored *stx* genes, previously confirmed by PCR [7]. Moreover, the strains analyzed belong to six different sequence types (STs): ST442, ST297, ST1131, ST2217, ST2387, and ST2520 [28]. Genomes of non-pathogenic/commensal strains available in databases were used as controls: *E. coli* HS (NCBI Assembly Refseq accession code GCF_000017765.1), *E. coli* DH5α (GCF_002899475.1), *E. coli* K-12 (GCF_000005845.2), *E. coli* MG1655 (GCF_000005845.2), *E. coli* K-12 (GCF_000019425.1), *E. coli* DH10B (GCF_000019425.1), and *E. coli* BL21 (GCF_000022665.1).

### 2.2. Comparative Analysis: Development of the Study Matrix

A total of 35.828 *E. coli* genomes were obtained from the Assembly Refseq database available in the National Center for Biotechnology Information (NCBI) of the United States of America (https://www.ncbi.nlm.nih.gov/genbank/, accessed on 8 January 2024) [29]. *E. coli* strains were classified as STEC or non-STEC group based on the presence or absence of any of the genes encoding Shiga toxin subunits (genes encoding A and B subunits of variants *stx1a*, *stx1c*, *stx2a*, *stx2b*, *stx2c*, *stx2d*, *stx2e*, *stx2f*, and *stx2g* were included). Non-STEC includes other pathotypes different from STEC.

The STs corresponding to each strain were identified using MLST [30], and genomes associated with the six above-mentioned STs were retrieved from the database. BUSCA (https://busca.biocomp.unibo.it/, accessed on 1 September 2024) was employed to predict protein subcellular localization specific to STEC [31]. A comparative genomic analysis based on the genetic content was performed using the Large-scale blast score ratio tool (LS-BSR) (available at https://github.com/jasonsahl/LS-BSR, accessed on 8 January 2024) [32]. Thus, the genetic content of all STEC strain genomes was compared to the genetic content of non-pathogenic *E. coli* strains *E. coli* HS, *E. coli* DH5α, *E. coli* K-12 MG1655, *E. coli* K-12 DH10B, and *E. coli* BL21. For each gene, genomic sequences with BSR ≥ 0.8 were considered positive, and those with BSR <0.4 were considered negative [32] (Supplementary Table S3). The LS-BSR pipeline generated a binary presence/absence matrix for 35,828 complete genomes. Detailed strain information and accession numbers are provided in the Supplemental (Supplementary Table S1).

### 2.3. Selection of Proteins of Study

Based on the matrix generated in Table S3, the fifteen most prevalent genes encoding uncharacterized proteins were selected. These genes were absent from commensal *E. coli* strains (BSR < 0.4) and were prioritized for evaluating their potential application as diagnostic markers.

To characterize the selected targets as proteins and to confirm that they encode conserved, structurally coherent proteins suitable as discriminatory genomic markers, several bioinformatic tools were used to analyze their sequences and predicted structures.

Functional annotation and structural prediction were performed through a consensus pipeline integrating multiple publicly available databases and web-based tools. Sequence similarity was evaluated by BLASTP 2.17.0+ (https://blast.ncbi.nlm.nih.gov/Blast.cgi?PROGRAM=blastp&PAGE_TYPE=BlastSearch&LINK_LOC=blasthome, accessed on 4 November 2024) against the UniProt (https://www.uniprot.org/blast, accessed on 4 November 2024) and NCBI nr databases to identify homologs and infer preliminary functions [33,34]. Conserved domains and protein families were characterized using InterProScan (https://www.ebi.ac.uk/interpro, accessed on 3 January 2025), CDD (https://www.ncbi.nlm.nih.gov/Structure/cdd/wrpsb.cgi, accessed on 15 September 2025), and HHpred (https://toolkit.tuebingen.mpg.de/tools/hhpred accessed on 20 September 2025) to define domain composition and superfamily relationships [35,36].

TMHMM 2.0 (https://services.healthtech.dtu.dk/services/TMHMM-2.0/, accessed on 16 September 2025) was used to predict transmembrane helices [37], and AlphaFold (https://alphafold.ebi.ac.uk/, accessed on 15 July 2025) to generate three-dimensional structural models supporting functional inference [38]. Protein–protein interaction networks were analyzed with STRING (https://string-db.org/, accessed on 16 July 2025) to explore functional associations based on genomic context, co-expression, and experimental evidence [39].

Expasy’s ProtParam server (https://web.expasy.org/protparam/, accessed on 16 July 2025) to calculate physicochemical parameters, including molecular weight, theoretical pI, charged residues, molecular formula, and instability index [40]. Finally, B-cell epitopes were predicted using the IEDB B-cell Epitope Prediction tool (https://tools.iedb.org/bcell/, accessed on 1 September 2025). Specifically, the BepiPred-2.0 algorithm was applied with default parameters [41]. This enables us to evaluate the potential of the candidate as a possible diagnostic target.

### 2.4. Statistical Analysis

The association between group membership (STEC vs. non-STEC) and the presence/absence of each of the most prevalent genes that codified proteins was assessed using Pearson’s Chi-square test (χ^2^). Yates’ correction was applied where appropriate. Results were considered statistically significant at a level of a *p*-value of <0.05. Additionally, the strength of the association was quantified by calculating the Odds Ratio (OR) with its corresponding 95% confidence interval (CI). The statistical analyses were performed using Graphpad and MedCalc (https://www.graphpad.com/quickcalcs/contingency1/, accessed on 1 November 2024 and https://www.medcalc.org/en/calc/odds_ratio.php, accessed on 1 January 2026, respectively). To evaluate the discriminative ability between STEC and non-STEC strains, a Receiver Operating Characteristic (ROC) curve analysis was performed. This analysis was used to determine the Area Under the Curve (AUC), along with its 95% confidence interval (CI), as a measure of overall predictive performance. The optimal cut-off point was determined by maximizing both sensitivity and specificity. Analyses were performed using Roc Analysis software (https://englab.rad.jhmi.edu/jrocfit/JROCFITi.html, accessed on 1 December 2025).

## 3. Results

Among the 35,828 RefSeq *E. coli* genomes, 247 were identified as ST297 (107 genomes), ST442 (100 genomes), ST2520 (22 genomes), ST2217 (9 genomes), ST2387 (7 genomes) or ST1131 (2 genomes), representing STs found in STEC non-O157 strains isolated in Argentina. After a comparison of the genetic content against non-pathogenic *E. coli* genomes, 947 STEC unique genes were identified, from which 113 encoding putative outer membrane or extracellular proteins were selected. These genes were screened in the whole database (35,828 genomes), which included 4980 STEC genomes (14%) and 30,848 as non-STEC. Among the most prevalent protein-coding genes within the STEC group, the most prevalent genes, or their encoded proteins, were selected for further analysis. A total of 15 were chosen for an in-depth characterization (Table 1).

From the initial set of candidates, five were excluded from the statistical analysis. Specifically, one showed cytoplasmic localization (number 1), two encoded Shiga toxin (numbers 12 and 13), one encoded an integrase (number 6), and one was of phage-related origin (number 14) (Table 1) (Supplementary Table S2). The ten remaining proteins showed significant association with STEC strains (*p* < 0.0001). However, both the magnitude of association (χ^2^) and the discriminative power (ROC AUC) varied among them (Table 1).

Three genes exhibited the strongest associations and the highest discriminatory capacities to detect STEC genomes, suggesting their potential as robust markers. One encoding a hypothetical protein (number 10: NCBI protein accession code WP283570181.1, χ^2^ = 14,633.7; OR: 43.66; AUC = 0.95), and two encoding putative uncharacterized hydrolases (number 4: NCBI protein accession WP_085453571.1; χ^2^ = 5454.483, OR: 23.81; AUC = 0.902 and number 15 NCBI protein accession code WP_249926694.1 χ^2^ = 9924.6; OR: 23.72; AUC = 0.906) (Table 1). Although protein number 10 showed the strongest statistical signal, it encodes a hypothetical protein, which makes it unsuitable as a biomarker. A blastn-based screening showed that genes encoding both hydrolases (4 and 10, Table 1) hit the same locus in a set of representative genomes, indicating that they were identified separately, but the gene is present only in single copies per genome. In addition, considering that proteins 4 and 15 share 81% identity and 88% sequence similarity, only one was prioritized as a potential marker. Protein 15 was ultimately selected for in-depth study based on its superior predictive performance, as indicated by a higher AUC value.

The hydrolase identified in this study showed a prevalence of 81.6% within the STEC group, while appearing in only 15.8% of non-STEC genomes. The distribution of genetic markers within the STEC group was evaluated, focusing on *eae* and *hes* as representative markers for LEE-positive and LEE-negative strains, respectively [19,42]. The *eae* gene and its variants (α, β, γ, δ, ε) were identified between 50.8% to 65.2% of the STEC group, while the *hes* gene was detected in 16.9%.

The hydrolase identified is composed of 472 amino acids. The prediction of the localization by BUSCA indicated that the extracellular space and its approximate molecular mass are ~52.19 kDa (Supplementary Table S2).

**Figure 1:**
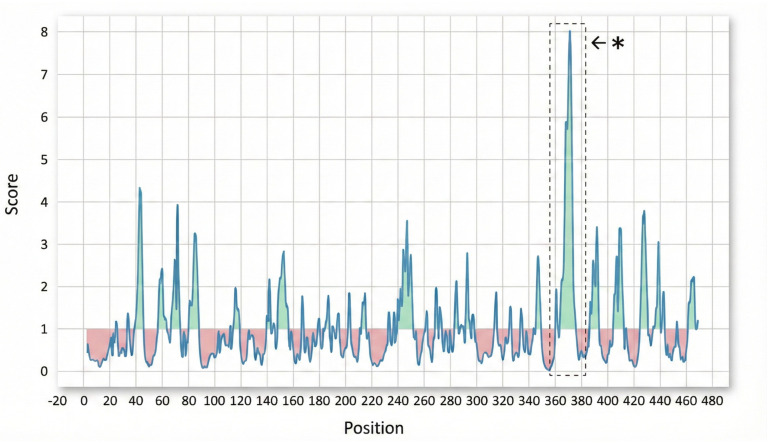
The graph illustrates the prediction score distribution across the amino acid sequence (*X*-axis: Position). Regions showing high peaks (green and pink spikes rising above the mean line) correspond to areas of the protein with the highest probability of being B-cell epitopes. * Highest-scoring epitopes.

The sequence was analyzed using BLAST, UniProt, InterProScan, HHpred, CDD, STRING, and AlphaFold. These analyses confirmed that the protein belongs to the Hydrolase family (Supplementary Table S2). InterProScan analysis identified an α/β-hydrolase fold (IPR029058), a highly conserved structural core common to hydrolytic enzymes of diverse functions, suggesting the protein may indeed act as a hydrolase. Epitope prediction analyses indicated the presence of several regions with the potential to act as B-cell epitopes (Figure 1). Among them, the peptide spanning residues 364 to 376 showed the highest predicted score (8.401), making it the most promising B-cell epitope candidate (Table 2).

**Figure 2:**
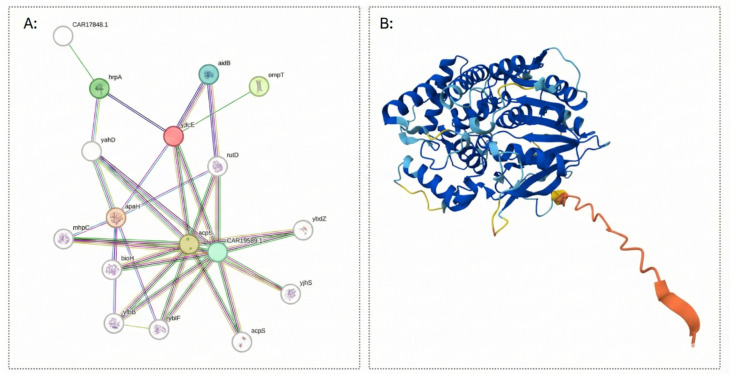
(**A**). STRING interaction network of the WP_249926694.1 protein. STRING analysis of the *E. coli* protein YlcE shows its predicted interaction partners. YlcE (red node) is connected to proteins of known or predicted function, including AidB, OmpT, ApaH, HrpA, AcpP. (**B**). Predicted three-dimensional structure of WP_249926694.1 based on its closest homolog (AF-A0A085NW94-F1-v6) obtained from AlphaFold. The color scale represents the confidence of the prediction, with blue indicating high-confidence regions and red representing lower-confidence regions.

Moreover, structural modeling using AlphaFold analysis revealed a 99% sequence identity (E-value = 0) with the α/β-hydrolase structure AF-A0A085NW94-F1-v6, confirming the conservation of the α/β-hydrolase fold and supporting the predicted hydrolase function of WP_249926694.1 (Figure 2). Furthermore, STRING analysis revealed that WP_249926694.1 is functionally associated with several extracellular or membrane-related proteins, including OmpT, AidB, and ApaH, among others. The functional network (Figure 1) shows that this hydrolase is part of a conserved functional module; its connectivity and co-occurrence suggest that it is a stable component of the STEC proteome.

## 4. Discussion

Reducing the zoonotic risk associated with STEC would significantly improve public health by enhancing food safety. Considering both the zoonotic and economic impact of STEC infections, along with the increasing number of cases worldwide [4], emphasizes the importance of diagnostic tools capable of detecting a broad range of STEC strains [43]. Although Shiga toxins (Stx) are the main virulence determinants in STEC, other virulence factors contribute to their pathogenicity. However, many of these cannot yet be considered reliable markers because they are not consistently present across the extensive genomic diversity of STEC (e.g., *eae*, *saa*, *ehxA*, *hes*) [19,42,44]. As a result, defining a STEC strain as pathogenic is complex/challenging since no combination of markers predicts the potential of a STEC strain to cause human disease. In LEE-positive strains, *eae* is a well-established predictor to detect pathogenic strains [42]. In contrast, for LEE-negative strains, various pathogenicity islands and virulence-associated genes have been described; however, their prevalence is generally below 54%, limiting their diagnostic usefulness (e.g., LAA, *hes*) [19,28].

In this study, the final selection of candidates was guided not only by their differential prevalence between STEC and non-STEC genomes, but also by biological criteria relevant to their potential diagnostic application. Accordingly, proteins predicted to be cytoplasmic were excluded due to their limited accessibility, which reduces their suitability as diagnostic targets [27]. Shiga toxin subunits were also excluded to avoid redundancy with well-established STEC markers and to prioritize the identification of alternative targets that may complement existing diagnostic approaches. In addition, proteins of phage origin or annotated as hypothetical in public databases were excluded because of their uncertain functional relevance, limited annotation support, and potential variability across strains, which could compromise their robustness and specificity as diagnostic markers. Collectively, these selection criteria allowed the analysis to focus on proteins with greater biological coherence and higher potential as reliable diagnostic markers for STEC.

A comprehensive in silico analysis identified two putative hydrolase genes, strongly associated with STEC strains. The statistical association observed, characterized by odds ratios of 23.72 and 23.81, and a χ^2^ value of 9924.59 and 5454.483 (*p* < 0.0001), demonstrates that the presence of these genes is a highly reliable indicator of the STEC pathotype. Although the hydrolase-encoding genes were identified separately, single hits were detected per positive genome, indicating that each strain harbors a single copy. These results, along with the high discriminatory power demonstrated in the ROC analysis, establish this protein as a reliable diagnostic target in STEC. Notably, this protein was present in 81.6% of STEC genomes, a statistically significant frequency compared to other potential markers analyzed in this study (such as *eae* and *hes*), regardless of the presence or absence of the LEE locus. When Galarce et al. [45] analyzed STEC genomes from South America, they reported marked variability in the prevalence of classical virulence-associated markers, with *eae* detected in only 30.8% of strains, *ehxA* in 60%, *saa* in 40.8%, and the LAA pathogenicity island in 43.1%. In this context, the markedly higher distribution of the hydrolase identified in the present study across diverse STEC lineages underscores its potential relevance as a broadly represented genomic marker.

The presence of the hydrolase was analyzed in the non-STEC group, where it was identified in 15.8% of the sequences. These results may be attributed to the significant genomic plasticity of *E. coli* strains, which is reflected in their highly diverse virulence profiles [46]. Although the hydrolase is not exclusively restricted to STEC, this does not diminish our findings, as the detection of this candidate marker is proposed as a complementary tool alongside detection of other genetic markers. The gene that codifies this hydrolase could be incorporated into existing multiplex PCR systems that differentiate *stx1* (180 bp), *stx2* (255 bp), and *eae* (384 bp) by amplicon length [42]. Preliminary primer designs (~100 bp) have already been generated for this purpose (data not published), which could facilitate the inclusion of its gene in epidemiological fast surveillance. Moreover, the hydrolase was detected across the six STs analyzed, indicating that the identified protein is not specific to a single lineage but is instead present in multiple genetic backgrounds within STEC. Functional and structural predictions obtained through multiple bioinformatic tools indicated that this protein belongs to the Hydrolase family, suggesting an enzymatic role related to bacterial metabolism or host interaction. The hydrolase class encompasses a wide range of enzymes that catalyze hydrolytic reactions using water to break chemical bonds, including proteases, lipases/phospholipases, glycosidases, and esterases [47].

The analysis performed with AlphaFold indicated a 99% structural association between WP_249926694.1 and the α/β-hydrolase AF-A0A085NW94-F1-v6, confirming the prediction obtained with the other software tools. Previous studies have reported that proteins belonging to the α/β-hydrolase family share a highly conserved, simple, and stable structural fold that supports the formation of diverse catalytic triads capable of mediating hydrolytic reactions [48]. While the general function, hydrolysis catalysis, can be inferred from the fold architecture and the presence of the catalytic triad, substrate specificity cannot be reliably predicted through in silico analysis alone. This limitation arises because minimal variations in the residues of the substrate-binding pocket can profoundly alter specificity. Therefore, accurate identification of the physiological substrate requires experimental validation or, alternatively, the analysis of conserved structural motifs within the binding pocket [48,49]. Moreover, STRING analysis associated the potential marker with several extracellular or membrane-related proteins, including OmpT, AidB and ApaH, which could be implicated in host interaction, stress responses, and virulence processes (Figure 1) [25,47,50,51].

Previous studies have demonstrated the role of hydrolases as virulence factors in other pathogens such as *Clostridium perfringens*, *Streptococcus pyogenes*, and *Staphylococcus aureus* [52,53,54]. The identified hydrolase in STEC could therefore be involved in virulence mechanisms, although its role remains to be experimentally validated.

The high-scoring epitope (364–376), together with other predicted epitopic regions identified in this protein, represents a promising target for the development of peptide-based or recombinant vaccines. The presence of multiple B-cell epitope candidates further suggests that combining these peptides may broaden immune coverage and enhance vaccine efficacy [55]. In addition to its potential as a vaccine antigen, the strong predicted humoral immunogenicity of these epitopic regions indicates that this protein could also serve as a valuable marker for serological assay development, improving both the sensitivity and specificity of current diagnostic approaches.

Despite the robustness of our comparative genomics approach, this study has certain limitations. First, as an exclusively in silico analysis, our findings regarding the expression, extracellular localization, and enzymatic activity of the candidate hydrolase require further experimental validation through in vitro assays. While the identified B-cell epitopes show high prediction scores, their actual immunogenicity and ability to elicit a protective response remain to be characterized. Additionally, the presence of the hydrolase gene in a fraction of non-STEC genomes suggests that, while it is a highly prevalent marker within STEC, its use in diagnostic frameworks should be complemented with traditional markers, such as Shiga toxin genes, to ensure maximum specificity. Future investigations should focus on characterizing its biochemical activity, confirming its expression in vitro, PCR validation, protein expression, and assessing the immunogenic potential of its predicted epitomic regions.

This study identifies, for the first time, a highly prevalent and previously uncharacterized extracellular hydrolase as a robust STEC marker with significant diagnostic and vaccine potential. Our findings offer a more comprehensive approach for identifying STEC strains in complex epidemiological scenarios and lay the groundwork for future experimental studies to characterize their biological role and validate their utility in public health surveillance and disease prevention.

## Figures and Tables

**Table 1:** Candidate protein-coding genes evaluated as potential STEC markers: localization, prevalence, and statistical validation.

N°	NCBI Accession	Length (aa)	Location	Functional Name	Prevalence	Χ^2^	OR (95% CI), *p* Value	Fitted ROC Area
1	WP_136816566.1	155	plasma membrane	Prepilin peptidase	4449/4980	*-	-	-
2	WP_108919521.1	696	extracellular space	Bifunctional siderophore receptor/adhesin Iha	3401/4980	χ^2^ = 4885.538	8.24 (7.71 to 8.80), *p* < 0.0001	0.801
3	AWM66617.1	105	outer membrane	Hypothetical protein	4464/4980	χ^2^ = 11,494.595	138,964.28 (8685.74 to 2,223,307.61), *p* < 0.0001	0.883
4	WP_085453571.1	494	extracellular space	Hydrolase	4077/4980	χ^2^ = 5454.483	23.81 (22.02–25.75), *p* < 0.0001	0.902
5	WP_308733525.1	243	extracellular space	TraT complement resistance	3916/4980	χ^2^ = 6260.662	2.71 (2.53–2.92), *p* < 0.0001	0.515
6	RDP83843.1	59	outer membrane	Probable site-specific recombinase/integrase	3488/4980	-	-	-
7	WP_407235464.1	190	extracellular space	Fimbrial protein	2648/4980	χ^2^ = 411.148	1.85 (1.74–1.97), *p* < 0.0001	0.623
8	WP_100008035.1	243	extracellular space	Putative lipoprotein	4192/4980	χ^2^ = 626.068	2.68 (2.48–2.90), *p* < 0.0001	0.811
9	OEI27840.1	93	outer membrane	Hypothetical protein	3092/4980	χ^2^ = 3121.57	10.48 (9.81 to 11.19), *p* < 0.0001	0.801
10	WP_283570181.1	59	extracellular space	Hypothetical protein	3300/4980	χ^2^ = 14,633.726.	43.66 (40.29 to 47.32), *p* < 0.0001	0.95
11	WP_001470661.1	63	outer membrane	DUF4222_uncharacterized	3623/4980	χ^2^ = 6392.410	11.44 (10.68–12.25), *p* < 0.0001	0.84
12	WP_210177975.1	89	extracellular space	Shiga-like toxin, beta subunit	3451/4980	-	-	-
13	WP_097746304.1	87	extracellular space	Shiga toxin Stx2b subunit B	3449/4980	-	-	-
14	SQQ40187.1	83	outer membrane	Putative prophage protein/hypothetical	3641/4980	-	-	-
15	WP_249926694.1	472	extracellular space	Aβ-hydrolase_1 (putative functional module)	4068/4980	χ^2^ = 9924.587, *p* < 0.0001	23.72 (21.94–25.65), *p* < 0.0001	0.906

NCBI accession: *RefSeq or GenBank* accession number corresponding to the protein sequence in public databases. Length (aa) Location: predicted subcellular localization (e.g., extracellular space, outer membrane, plasma membrane), determined using BUSCA and complementary tools. Predicted function: functional annotation inferred from BLAST, InterProScan, UniProt, and HHpred analyses (e.g., hydrolase, lipoprotein, fimbrial protein, complement resistance factor). Prevalence: number of STEC genomes (out of 4980) in which the gene was detected. Chi^2^: Chi-square statistic evaluating the association between gene presence and STEC status (all *p* < 0.0001). OR: Odds Ratio; 95% CI, 95% Confidence Interval. Fitted ROC area: area under the ROC curve estimating the discriminatory capacity of each gene to differentiate STEC genomes (AUC closer to 1 indicates better performance). *- Statistical analysis was not performed.

**Table 2:** Candidate B-cell epitopes predicted from the protein sequence.

Start	End	Peptide	Length	Score
35	40	QERRPD	6	1.833
60	68	KYYTDTS	9	1.968
85	93	TLKWWKS	9	1.944
113	119	SGLYQVA	7	1.94
143	148	WETRDSRY	6	1.98
202	208	QLKAQPD	7	1.921
240	252	QLTEPDSRKDA	13	1.916
364	376	SLTGEEDSSKLDA	13	8.401
387	393	DRKGSGVS	7	1.838
402	408	QRGQSYS	7	1.86
420	426	QQSYTS	7	1.871
451	457	QEPSPSL	7	1.885

Start: N-terminal residue position of the predicted peptide segment in the full protein sequence. End: C-terminal residue position of the predicted peptide segment in the full protein sequence. Peptide: The amino acid sequence of the predicted epitope candidate. Length: The number of amino acid residues in the predicted epitope segment (End − Start + 1). Score: The aggregated prediction score for the peptide segment, representing the confidence level of its potential antigenicity. Higher scores indicate a greater likelihood of being a functional B-cell epitope.

## Data Availability

The original contributions presented in this study are included in the article/Supplementary Materials. Further inquiries can be directed to the corresponding author.

## Supplementary Materials

The following supporting information can be downloaded at https://www.mdpi.com/article/10.3390/vetsci13020153/s1. Table S1: Comprehensive genomic dataset and presence/absence matrix of virulence factors across 35,828 *Escherichia coli* strains. Table S2: Comprehensive bioinformatic profiling of candidate markers: results from functional, structural, and localization analysis tools. Table S3: Specificity cross-screening: sequence identity of candidate markers against commensal and laboratory Escherichia coli reference strains.

## Abbreviations

The following abbreviations are used in this manuscript:

*E. coli*	*Escherichia coli*
STEC	Shiga toxin-producing *Escherichia coli*
Stx	Shiga toxin
LEE	Locus of Enterocyte Effacement
HUS	Hemolytic Uremic Syndrome
OMP(s)	Outer membrane protein(s)
ST(s)	Sequence type(s)
MLST	Multilocus sequence typing
LS-BSR	Large-scale BLAST score ratio
BSR	BLAST score ratio
ROC	Receiver Operating Characteristic
AUC	Area under the curve
CI	Confidence interval
χ^2^	Chi-square test
TMHMM	Transmembrane Hidden Markov Model
